# Idiopathic male infertility in the Han population in China is affected by polymorphism in the VDAC2 gene

**DOI:** 10.18632/oncotarget.12993

**Published:** 2016-10-31

**Authors:** Lianjun Pan, Daoxian Qiu, Jingyun Li, Jun Li, Pu Xu, Dan Zhao, Jiehua Ma

**Affiliations:** ^1^ State Key Laboratory of Reproductive Medicine, Department of Urology, Nanjing Maternity and Child Health Care Hospital Affiliated to Nanjing Medical University, Nanjing 210004, China; ^2^ Department of Urology, Yidu Central Hospital of Weifang, Qingzhou 262500, China; ^3^ State Key Laboratory of Reproductive Medicine, Department of Plastic and Cosmetic Surgery, Nanjing Maternity and Child Health Care Hospital Affiliated to Nanjing Medical University, Nanjing 210004, China; ^4^ Department of Gynaecology, Nanjing Maternity and Child Health Care Hospital Affiliated to Nanjing Medical University, Nanjing 210004, China

**Keywords:** VDAC2, genetic diversity, semen parameters, idiopathic male infertility

## Abstract

**Background:**

It has been proved that human voltage-dependent anion channel 2 (VDAC2) plays a significant role in sperm function and male fertility. This study was primarily aimed at exploring whether VDAC2 is a risk factor for idiopathic male infertility.

**Results:**

We determined a significantly increased risk of idiopathic infertility with abnormal semen parameters in association with the variant rs2804535 and a decreased risk of idiopathic infertility with abnormal semen parameters in association with the variant rs11001334. However, among subjects with normal semen parameters, no significant differences could be found in these genotypes. Moreover, we could not find any differences in the variants rs7896741 and rs1259503, which showed no risk of male infertility, whether normal or abnormal.

**Materials and methods:**

All of the experimental subjects, including 523 men who cannot conceive children and 277 fertile controls, underwent complete historical and physical examinations. Each participant donated an ejaculate for semen analysis and 5 ml of peripheral blood for genomic DNA extraction. A computer-assisted semen analysis system was used for the semen analysis. Four single-nucleotide polymorphisms were identified and analyzed using TaqMan SNP Genotyping Assays.

**Conclusions:**

The result shows that the relationships between different variants in the VDAC2 gene and male fertility differ, and the individuals who carry those variants may have a decreased or increased risk of abnormal semen parameters associated with male infertility.

## INTRODUCTION

Studies have been conducted on voltage-dependent anion channel (VDAC) proteins, i.e., channel-forming proteins in the outer mitochondrial membrane (OMM) because of their protective action in modulating metabolite exchange and the transfer of ATP as well as ions between the cytoplasm and mitochondria [1]. There are three isoforms of VDAC, VDAC1, VDAC2 and VDAC3, which show >70% similarity in their sequence. Even though all VDAC isoforms can appear everywhere, the major form is expressed in sperm outer dense fibers (ODFs), an important separate part of the cytoskeleton without a membrane that is involved in flagellar motility [2]; ODFs are mainly focused on the top of the membrane and principal piece of the sperm [3–5]. These proteins also play roles as transportation proteins in sperm functions by mediating transmembrane calcium transport [4]. A recent study showed that VDAC2 mRNA tends to be more enriched in less motile sperm [6]. A proteomic study revealed that VDAC2 has a strong relationship with male fertility [7]. On the other hand, it is still unknown that whether differences in VDAC2 increase the risk of male fertility or not.

The VDAC2 gene encodes human VDAC2, which is also named outer mitochondrial membrane protein porin 2 (UniProtKB ID: P45880; Genetic ID: 7417). This gene is approximately 20.6 kb in length, is located on chromosome 10q22.2 and includes 13 exons [8]. Using HapMap genotype data, which were obtained from Han Chinese subjects who live in Beijing, four tag single-nucleotide polymorphisms (tagSNPs) (rs2804535, rs7896741, rs11001334 and rs1259503) were selected. Then, we began the analysis of those four genotyping-identified tagSNPs in the VDAC2 gene and measured how those genetic variants affect semen parameters with respect to male infertility; however, all steps were completed with the help of the hospital, which recruited 523 men with infertility and 277 fertile men from the Han-Chinese population.

## RESULTS

The mean age of the 523 males in the Han population in this study who could not have babies was 32.0 ± 5.6 years of age (range 20–55 years). The time between experimentation and semen collection was 5.0 ± 2.7 days (range 2–20 days). In addition, the sperm parameters are presented in Table 1. Briefly, there was nothing uncommon or special in the stratification for smoking and drinking for all the features above. However, the sperm number per ejaculate, sperm concentration, and sperm motility exhibited significant differences among the three age groups (*P* = 0.001, 0.001 and 0.034, respectively). The sperm number per ejaculate and sperm concentration were significantly greater in the 25 kg/m^2^ group than the < 25 kg/m^2^ group (*P* = 0.036 and 0.028, respectively). In regard to the duration of sexual abstinence, the ≥7 days group had a higher semen volume and sperm number per ejaculate than the < 7 days group (*P* = 0.000 and 0.001, respectively). The remaining parameters (sperm concentration, sperm number per ejaculate, sperm motility, and semen volume) were similar after stratification for age, BMI and the duration of sexual abstinence.

**Table 1:** Association between the selected individual characteristics and sperm parameters in 523 males with definite idiopathic infertility

	N (%)	Semen volume (mL)	Concentration (× 10^6^/mL)^a^	Sperm number per ejecalate (×10^6^/ejaculum)^a^	Motility (%)
**Age (years)**					
≤ 29	183 (34.99)	3.27 ± 1.35	**3.49 ± 1.28^b^**	**4.63 ± 1.41^b^**	**43.70 ± 26.32^b^**
29–33	183 (34.99)	3.39 ± 1.41	**3.80 ± 1.17**	**4.97 ± 1.21**	**49.72 ± 26.04**
> 33	157 (30.02)	3.39 ± 1.27	**3.95 ± 0.95**	**5.11 ± 1.02**	**49.65 ± 21.98**
**Smoking**					
Yes (ever)	260 (49.71)	3.31 ± 1.22	3.71 ± 1.16	4.87 ± 1.21	48.12 ± 24.45
No (never)	263 (50.29)	3.38 ± 1.47	3.79 ± 1.16	4.94 ± 1.26	47.07 ± 25.78
**Drinking**					
Yes (ever)	207 (39.58)	3.30 ± 1.36	3.66 ± 1.25	4.81 ± 1.33	45.32 ± 26.20
No (never)	316 (60.42)	3.37 ± 1.34	3.80 ± 1.09	4.97 ± 1.18	49.08 ± 24.30
**BMI**					
< 20	49 (9.37)	3.25 ± 1.22	**3.46 ± 1.24^b^**	**4.58 ± 1.28^b^**	44.70 ± 27.61
20–25	293 (56.02)	3.33 ± 1.36	**3.70 ± 1.19**	**4.85 ± 1.30**	46.98 ± 24.53
≥ 25	181 (34.61)	3.40 ± 1.37	**3.90 ± 1.07**	**5.07 ± 1.10**	49.37 ± 25.35
**Abs days**					
< 4	190 (36.33)	**2.88 ± 1.10^b^**	3.63 ± 1.13	**4.65 ± 1.18^b^**	49.50 ± 25.74
4–7	203 (38.81)	**3.49 ± 1.34**	3.76 ± 1.10	**4.97 ± 1.22**	48.26 ± 24.36
≥ 7	130 (24.86)	**3.81 ± 1.49**	3.91 ± 1.28	**5.18 ± 1.29**	43.77 ± 25.12

Values are mean ± SD unless otherwise stated.

BMI = body mass index; Abs = the duration of sexual abstinence.

a Values were re-transformed following Logarithmic transformation.

b *P* < 0.05 for comparison between groups using analysis of variance (ANOVA).

Table 2 illustrates the sperm parameters and the genotype frequency for each variant. The relationship between the sperm number per ejaculate and the subjects was not clear. The sperm concentration, semen volume, and sperm motility were largely similar between the genotypes of the two variants (rs2804535 and rs7896741). For rs11001334, the sperm concentration in subjects with the TT genotype was significantly lower than that in those harboring the CC genotype (*P* = 0.045) and significantly higher in subjects with the CT and TT genotypes than those harboring the CC genotype (*P* = 0.037). For rs1259503, the semen volume in subjects with the GC and CC genotypes was significantly lower than that in the subjects harboring the GG genotype (*P* = 0.039) (Table 2). As shown in Table 3, we initially divided the cases into the case Group I and case Group II by semen parameters and found that no significant relationships were found for the frequency of all four genotype variants.

**Table 2:** Sperm parameters according to the genetic variants of *VADC2* gene in 523 males with definite idiopathic infertility

Variant	Genotype	N (%)	Semen volume (mL)	Concentration^a^	Sperm number per ejecalate^a^	Motility (%)
rs2804535	TT	443 (84.70)	3.38 ± 1.34	3.76 ± 1.14	4.94 ± 1.21	48.17 ± 25.38
	TC	80 (15.30)	3.16 ± 1.37	3.69 ± 1.24	4.73 ± 1.35	44.38 ± 23.48
	CC	0 (0.00)	-	-	-	-
	TC + CC	80 (15.30)	3.16 ± 1.37	3.69 ± 1.24	4.73 ± 1.35	44.38 ± 23.48
rs7896741	GG	300 (57.36)	3.36 ± 1.30	3.70 ± 1.23	4.86 ± 1.28	48.12 ± 24.22
	GC	193 (36.90)	3.36 ± 1.46	3.81 ± 0.99	4.98 ± 1.14	46.76 ± 25.90
	CC	30 (5.74)	3.06 ± 1.10	3.81 ± 1.37	4.88 ± 1.46	47.69 ± 29.20
	GC + CC	223 (42.64)	3.32 ± 1.41	3.81 ± 1.04	4.97 ± 1.18	46.89 ± 26.30
rs11001334	CC	453 (86.62)	3.34 ± 1.36	3.76 ± 1.15	4.91 ± 1.23	46.70 ± 25.48
	CT	69 (13.19)	3.34 ± 1.29	3.72 ± 1.21	4.88 ± 1.25	**53.91 ± 21.52^b^**
	TT	1(0.19)	4.20 ± 0.00	**1.61 ± 0.00^c^**	3.05 ± 0.00	16.38 ± 0.00
	CT + TT	70 (13.38)	3.36 ± 1.29	3.69 ± 1.23	4.85 ± 1.26	**53.37 ± 21.83^d^**
rs1259503	GG	194 (37.09)	3.49 ± 1.29	3.74 ± 1.13	4.94 ± 1.18	49.88 ± 24.82
	GC	250 (47.80)	**3.29 ± 1.41^e^**	3.79 ± 1.16	4.94 ± 1.24	45.73 ± 24.79
	CC	79 (15.11)	3.18 ± 1.28	3.62 ± 1.23	4.69 ± 1.35	47.86 ± 26.64
	GC + CC	329 (62.91)	**3.26 ± 1.38^f^**	3.75 ± 1.18	4.88 ± 1.27	46.24 ± 25.22

All *P*-values were adjusted for age, smoking, drinking, body mass index (BMI) and the duration of sexual abstinence (Abs).

Values are mean ± SD unless otherwise stated.

a Values are re-transformed following Logarithmic transformation.

b *P* < 0.05 for comparison between rs11001334 CT and CC genotype (*P* = 0.026).

c *P* < 0.05 for comparison between rs11001334 TT and CC genotype (*P* = 0.045).

d *P* < 0.05 for comparison between rs11001334 CT+TT and CC genotype (*P* = 0.037).

e *P* = 0.060 for comparison between rs1259503 GC and GG genotype.

f *P* < 0.05 for comparison between rs1259503 GC+CC and GG genotype (*P* = 0.039).

**Table 3:** Genotype frequencies of *VADC2* genetic variants among the cases and controls and their association with male infertility

SNPs	Genotype	Control	Case group 1			Case group 2		
		*N* (%)	*N* (%)	*P*	*OR*(95%*CI*)	*N* (%)	*P*	*OR*(95%*CI*)
rs2804535								
	TT	205 (85.06)	127 (89.44)		1.00	316 (82.94)		1.00
	TC	34 (14.11)	15 (10.56)	0.608	0.86 (0.50–1.51)	65 (17.06)	0.645	1.24 (0.79–1.95)
	CC	2 (0.83)	0			0		
	TC + CC	36 (14.94)	15 (10.56)	0.226	0.67 (0.35–1.28)	65 (17.06)	0.488	1.17 (0.75–1.83)
rs7896741								
	GG	143 (59.34)	81 (57.04)		1.00	219 (57.48)		1.00
	GC	85 (35.27)	51 (35.92)	0.781	1.06 (0.68–1.65)	142 (37.27)	0.714	1.09 (0.78–1.54)
	CC	13 (5.39)	10 (7.04)		1.36 (0.57–3.24)	20 (5.25)		1.01 (0.48–2.08)
	GC + CC	98 (40.66)	61 (42.96)	0.660	1.10 (0.72–1.67)	162 (42.52)	0.648	1.08 (0.78–1.50)
rs11001334								
	CC	197 (81.74)	119 (83.80)		1.00	334 (87.66)		1.00
	CT	44 (18.26)	23 (16.20)	0.608	0.87 (0.50–1.51))	46 (12.07)	0.069	0.83 (0.53–1.12)
	TT	0	0			1 (0.26)		
	CT + TT	44 (18.26)	23 (16.20)	0.608	0.87 (0.50–1.51)	47 (12.33)	0.062	0.80 (0.64–1.11)
rs1259503								
	GG	95 (39.42)	65 (45.77)		1.00	129 (33.86)		1.00
	GC	113 (46.89)	54 (38.03)	0.107	0.70 (0.44–1.10)	196 (51.44)	0.446	1.28 (0.90–1.82)
	CC	33 (13.69)	23 (16.20)		1.02 (0.55–1.89)	56 (14.70)		1.25 (0.75–2.07)
	GC + CC	146 (60.58)	77 (54.23)	0.224	0.77 (0.51–1.17)	252 (66.14)	0.160	1.27 (0.91–1.78)

Group 1: a + b > 50%, Semen volume (mL) > 2 ml, Concentration > 20 × 10^6^ml.

Group 2: Subjects consisted of idiopathic infertile men with at least one of the abnormal semen parameters (semen volume, sperm concentration, sperm number per ejaculum and sperm motility).

*P* values were obtained using two-sided χ2 test for genotype distributions between cases and controls.

ORs were obtained from multivariate logistic regression analysis.

As shown in Table 4, the rs2804535 genotype frequencies were 67.86% (TT), 32.14% (TC) and 0% (CC) in the cases of Subgroup 2 and 85.06% (TT), 14.11% (TC) and 0.83% (CC) in the controls, and they showed statistically significant differences (*P* = 0.023). A co-dominant allele effect might exist when we put the rs2804535 TC and the rs2804535 CC together, and we can also see a greater number of the rs2804535 TC + CC variant genotypes in the group (*P* = 0.025). Logistic regression analysis showed us that the frequency of the heterozygous rs2804535 TC genotype (OR = 2.86, 95% CI = 1.19–6.83) and the rs2804535 TC + CC variant genotypes (OR = 2.69, 95% CI = 1.13–6.43) was higher in the subjects in Subgroup 2. However, the frequency of rs2804535 was not higher in the other subgroups.

**Table 4:** Genotype frequencies of *VADC2* genetic variants among the cases and controls and their association with male infertility

SNPs	Genotype	control	Subgroup 1			Subgroup 2			Subgroup 3			Subgroup 4		
		*N* (%)	*N* (%)	*P*	*OR* (95%*CI*)	*N* (%)	*P*	*OR* (95%*CI*)	*N* (%)	*P*	*OR* (95%*CI*)	*N*(%)	*P*	*OR* (95%*CI*)
rs2804535														
	TT	205 (85.06)	294 (83.29)		1.00	19 (67.86)		1.00	115 (83.94)		1.00	93 (84.55)		1.00
	TC	34 (14.11)	59 (16.71)	NS	1.21 (0.77–1.91)	9 (32.14)	**0.023**	**2.86 (1.19–6.83)**	22 (16.06)	NS	1.15 (0.64–2.07)	17 (15.45)	NS	1.10 (0.59–2.07)
	CC	2 (0.83)	0			0			0			0		
	TC+CC	36 (14.94)	59 (16.71)	NS	1.14 (0.73–1.79)	9 (32.14)	**0.025**	**2.69 (1.13–6.43)**	22 (16.06)	NS	1.09 (0.61–1.94)	17 (15.45)	NS	1.04 (0.56–1.95)
rs7896741														
	GG	143 (59.34)	205 (58.07)		1.00	17 (60.71)		1.00	80 (58.39)		1.00	64 (58.18)		1.00
	GC	85 (35.27)	129 (36.54)	NS	1.06 (0.74–1.50)	10 (35.71)	NS	0.99 (0.43–2.26)	47 (34.31)	NS	0.99 (0.63–1.55)	37 (33.64)	NS	0.97 (0.60–1.58)
	CC	13 (5.39)	19 (5.38)		1.02 (0.49–2.13)	1 (3.75)		0.64 (0.08–5.26)	10 (7.30)		1.38 (0.58–3.28)	9 (8.18)		1.55 (0.63–3.80)
	GC + CC	98 (40.66)	148 (41.93)	NS	1.05 (0.76–1.47)	11 (39.29)	NS	0.94 (0.42–2.10)	57 (41.61)	NS	1.04 (0.68–1.59)	46 (41.82)		1.05 (0.66–1.66)
rs11001334														
	CC	197 (81.74)	311 (88.10)		1.00	25 (89.29)		1.00	119 (86.86)		1.00	96 (87.27)		1.00
	CT	44 (18.26)	41 (11.61)	**0.030**	**0.59 (0.37–0.96)**	3 (10.71)	NS	0.54 (0.16–1.86)	17 (12.41)	NS	0.64 (0.35–1.17)	13 (11.82)	NS	0.61 (0.31–1.18)
	TT	0	1 (0.28)			0			1 (0.73)			1 (0.91)		
	CT + TT	44 (18.26)	45 (11.89)	**0.032**	**0.61 (0.38–0.96)**	3 (10.71)	NS	0.54 (0.16–1.86)	18 (13.14)	NS	0.68 (0.37–1.23)	14 (12.73)	NS	0.65 (0.34–1.25)
rs1259503														
	GG	95 (39.42)	124 (35.13)		1.00	7 (25.00)		1.00	51 (37.23)		1.00	41 (37.27)		1.00
	GC	113 (46.89)	180 (50.99)	NS	1.22 (0.86–1.91)	15 (53.57)	NS	1.80 (0.71–4.60)	61 (44.53)	NS	1.01 (0.63–1.60)	50 (45.45)	NS	1.03 (0.63–1.68)
	CC	33 (13.69)	49 (13.88)		1.14 (0.68–1.91)	6 (21.43)		2.47 (0.77–7.87)	25 (18.25)		1.41 (0.76–2.63)	19 (17.27)		1.33 (0.68–2.62)
	GC + CC	146 (60.58)	229 (64.87)	NS	1.20 (0.96–1.69)	21 (75.00)	NS	1.95 (0.80–4.77)	86 (62.77)	NS	1.10 (0.71–1.69)	69 (62.73)	NS	1.10 (0.69–1.74)

Subgroup1: a + b < 50%; Subgroup2: semen volume < 2 ml; Subgroup3: sperm concentration < 20 × 10^6^ ml; Subgroup4: sperm number per ejaculum < 40 × 10^6^

*P* values were obtained using two-sided χ2 test for genotype distributions between cases and controls.

ORs were obtained from multivariate logistic regression analysis.

NS, not statistically significant.

Furthermore, the rs11001334 genotype frequencies were 88.10% (CC), 11.61% (CT) and 0.28% (TT) in the cases in Subgroup 1 and 81.74% (CC), 18.26% (CT) and 0% (TT) in the controls, with an obvious statistical difference (*P* = 0.030). When we combined rs11001334 CT and rs11001334 TT, there was a co-dominant allele effect, and the combination of the rs11001334 CT + TT variant was lower in the experimental group (*P* = 0.032). Logistic regression analysis indicated that the heterozygous rs11001334 CT genotype (OR = 0.59, 95% CI = 0.37–0.96) and the rs11001334 CT + TT variant genotype (OR=0.61, 95% CI=0.38–0.96) were present at remarkably lower frequency in the subjects in Subgroup 1. However, there was no evidence of rs7896741 and rs1259503 among the study and the experimental subjects.

## DISCUSSION

Although it has been suggested that human VDAC2 plays an important role in sperm function and male fertility [5, 7, 9, 10], there have been no related studies of the hidden role that VDAC2 plays in male infertility, particularly idiopathic male infertility. This research was primarily aimed at analyzing 4 different tagSNPs (rs2804535, rs7896741, rs11001334 and rs1259503) of the VDAC2 gene to determine their relationship with male infertility. For the variant rs11001334, we determined that there was a negative relationship between this SNP and sperm parameters. In addition, there was a positive relationship between the same parameters and variant rs2804535 (*P* = 0.030 and 0.023). However, the men with normal semen parameters shared the same genotypes. Furthermore, among the rs7896741 and rs1259503 variants, there were no signs of a relationship with the risk of male infertility. These results illustrated that the different polymorphisms in the VDAC2 gene are not equal because they have extremely different relationships (positive or negative) with unique semen parameters in infertile males.

The SNP location helps predict disease etiology [11]. Despite the fact that the most common polymorphisms are situated in exonic sequences, an increasing number are located in introns [12–16]. Recently, mutations deep in intronic sequences were reported to alter splicing efficiency by the means of disrupting or producing a splicing motif, affecting the binding of the splicing machinery, which could lead to a disorder in impacted individuals [12]. By analyzing the locations of the four tagSNPs (rs2804535, rs7896741, rs11001334 and rs1259503), we found that the SNP rs2804535 (T > C) was located within the fifth intron of the human VDAC2 gene. Thus, we hypothesized that the intronic SNP rs2804535 (T > C) may modulate the splicing efficiency of the VDAC2 gene. Specific motifs in the VDAC2 protein are required for mitochondrial Bak import [17, 18]. Therefore, this SNP may affect VDAC2 protein function by modulating the splicing efficiency of the VDAC2 gene and may ultimately result in idiopathic male infertility.

Interestingly, a study reported that one SNP in exon 5 of a non-coding RNA increased the transcriptional level of the novel gene and may play some role in making people sick [19]. In our study, the SNP rs11001334 (C > T) is a non-coding transcript exon variant located in exon 12 and may affect the transcriptional level of a non-coding RNA, which then may alter the transcription of the VDAC2 gene and may ultimately result in idiopathic male infertility. Furthermore, the rs11001334 (C > T) genotype had a strong relationship with sperm concentration and motility. Through our data, we confirmed that different polymorphisms in VDAC2 are responsible for the inability to conceive a baby along with abnormal semen parameters. The physiological impact of these SNPs on idiopathic male infertility requires further study.

## MATERIALS AND METHODS

### Subjects

This research was started with the approval of the Medical Ethics Committee of Nanjing Maternity and Child Health Care Hospital. From March 2014 to June 2015, 523 male patients who were proven to be infertile (while their wives were not) and 277 fertile men from the Nanjing Maternity and Child Health Care Hospital were continuously recruited to participate in the experiment. All the patients were of Han-Chinese ethnicity, had been informed of the aim of this study, and had been provided time to consider enrolling. Patients with idiopathic male infertility were asked to not to have sex or conceive a child for at least one year. Individuals who suffered from orchitis, congenital bilateral absence of the vas deferens, obstruction, cryptorchidism, Y chromosome microdeletions, and cytogenetic abnormalities were not ideal candidates for the study, as previously described [20]. The experimental subjects had already fathered at least one child without using any assistive techniques. All the patients provided a medical history and underwent physical examinations, which also required that they provide a volume of blood of 5 ml with the purpose of genomic DNA extraction as well as an ejaculate for performing semen analysis following a questionnaire.

### Semen analysis

The analysis of semen was carried out in a manner similar to what has been described previously [21].

There are four indexes that were used to evaluate the association with VDAC2 gene polymorphisms, including sperm concentration, semen volume, sperm motility and sperm number per ejaculate.

### VDAC2 SNP selection and genotyping

**Figure 1:**
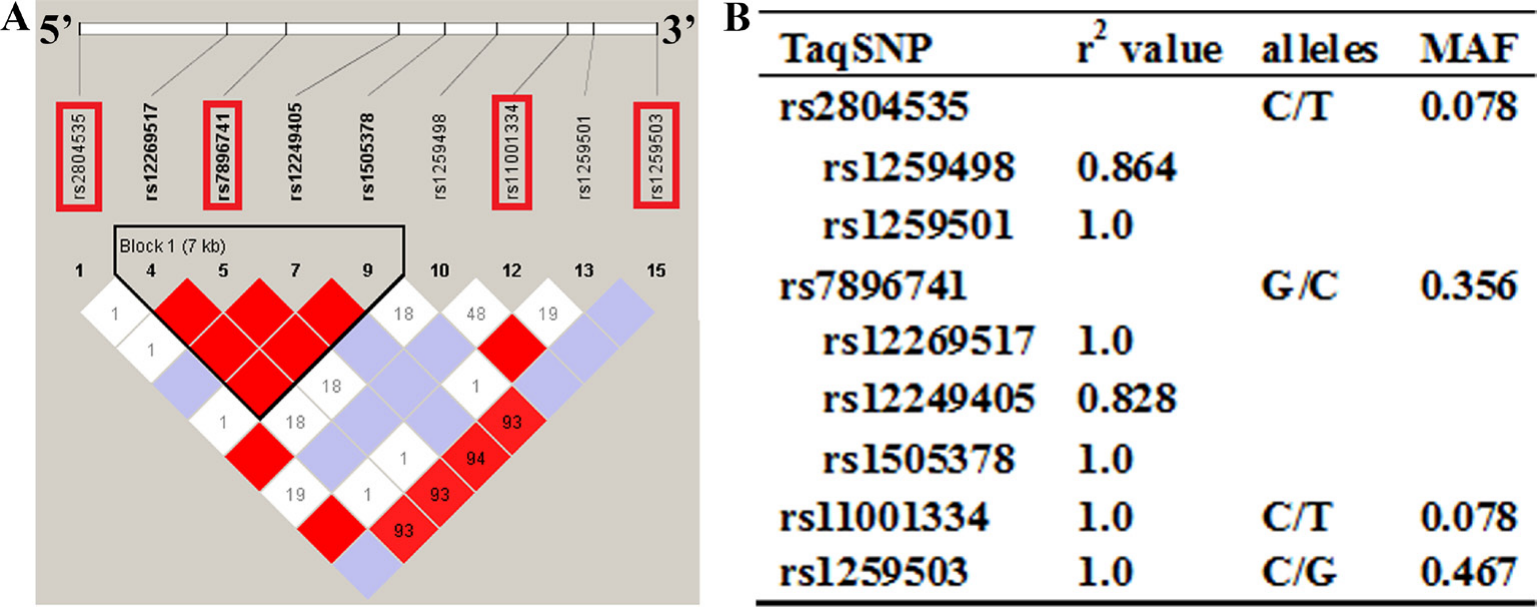
The overlook of the chosen tagging single nucleotide polymorphisms (tagSNPs) and the individual characteristics (**A**) Site of the four SNPs in the VDAC2 gene and 1500 base pairs (bp) upstream and 1500 bp downstream region. The selected tagSNPs are indicated by red frame. (**B**) TagSNPs are evaluated in a indirect way by the listed r2 values correspond. MAF = minor allele frequency.

Using the HapMap genotype data that were collected from non-related Han Chinese individuals from the city of Beijing (HapMap Data Rel 24/phase II nov08, on NCBI B36 assembly, dbSNP b126; http://hapmap.ncbi.nlm.nih.gov), SNPs within the 20641 bp human VDAC2 gene as well as the 1500 bp upstream and 1500 bp downstream that had a low frequency (>0.05%) in the Han Chinese population in Beijing and were located precisely on chromosome 10 from 76640569 to 76661210 were selected. Nineteen SNPs were identified in the region. Linkage disequilibrium (LD) in the zone was evaluated with the r2 values, which show the likelihood a certain SNP is capable of predicting the presence of other SNPs [22], using Haploview 4.0 software. By applying Tagger and a tagging threshold of r2 higher than 0.80, four tagSNPs (rs2804535, rs7896741, rs11001334 and rs1259503) that could predict the other SNPs from 76640569 to 76661210 were selected, with a mean r2 value of 1.0 (Figure 1).

Genomic DNA was isolated from peripheral blood leukocytes of the 523 Han Chinese men who suffered from the inability to conceive a baby in accordance with the prescribed protocols (Genomic DNA kit; Tiangen, Beijing, China). Using the Taq amplification method, TaqMan SNP Genotyping Assays were used for genotyping in an ABI Prism 7900 HT Fast Real-Time PCR system (Applied Biosystems, USA). The PCR amplification protocol included an initial step at 95°C for 10 minutes, then 40 cycles at the temperature of 95°C for approximately 15 seconds, 56°C for 10 seconds, and 60°C for 1 minute, and an additional step at 60°C for 10 minutes. Approximately 10 percent of the people who volunteered for the study were randomly chosen. In our study, the SNP genotyping coverage was perfect, as was the concordance of duplicates.

### Statistical analysis

The statistical analysis was successfully completed in the Stata statistical package (Version 7.0, StataCorp, LP). The online software SHEsis is very effective in determining Hardy-Weinberg equilibrium (HWE) (http://analysis.bio-x.cn/myAnalysis.php) [23]. Logarithmic transformation was performed for the sperm concentration to confirm a normal distribution, and the outlying data were excluded. Cut-offs for the semen parameters were established according to the WHO reference values (World Health Organization, 1999 World Health Organization; Cambridge, UK: Cambridge University Press, 1999; WHO Laboratory Manual for the Examination of Human Semen and Sperm-Cervical Mucus Interaction, 4th ed.) for sperm concentration (< 20 × 10^6^/ml), semen volume (< 2 ml), sperm motility (< 50% motile sperm), and sperm number per ejaculate (< 40 × 10^6^/ml). The experimental subjects who previously conceived a child were split into 2 groups: case Group I, defined as male-factor infertility that met all four parameters, while case Group II failed to meet one or more of the semen parameters and was further broken into four subgroups: Subgroup I (sperm motility < 50% motile sperm), Subgroup II (semen volume < 2 ml), Subgroup III (sperm concentration < 20 × 10^6^/ml) and Subgroup IV (sperm number per ejaculate < 40 × 10^6^/ml). In the case of Group II, each of the subjects may have been classified into more than one subgroup. The method of comparing to the mean age, body mass index (BMI) and duration of sexual abstinence was used to analyze the difference of variance in the case of Group II. The Mann-Whitney U test was used to attempt to determine the differences in the subjects' daily life behaviors, such as smoking and the frequency of the VDAC2 allele in case groups. Multivariate logistic regression analysis was conducted to find the odds ratios (ORs) in the proven infertile men and the 95 percent confidence intervals (95% CI), adjusting for age, smoking and drinking habits, and body mass index (BMI) when appropriate. The χ2 test was used to precisely identify the difference in selected VDAC2 alleles between the HapMap study project and the experimental subjects. Differences were considered significant at *P* < 0.05.
